# The pharmacokinetics profiles, pharmacological properties, and toxicological risks of dehydroevodiamine: A review

**DOI:** 10.3389/fphar.2022.1040154

**Published:** 2022-11-18

**Authors:** Shubin Fu, Liying Liao, Yi Yang, Yan Bai, Yan Zeng, Haoyu Wang, Jianxia Wen

**Affiliations:** ^1^ Jiujiang Inspection and Testing Certification Center, Jiujiang, China; ^2^ School of Food and Bioengineering, Xihua University, Chengdu, China

**Keywords:** dehydroevodiamine (DHE), *Evodiae fructus* (EF), pharmacokinetics profiles, pharmacological properties, toxicological risks

## Abstract

Dehydroevodiamine (DHE) is a quinazoline alkaloid isolated from *Evodiae Fructus* (EF, *Wuzhuyu* in Chinese, Rutaceae family), a well-known traditional Chinese medicine (TCM) which is clinically applied to treat headache, abdominal pain, menstrual pain, abdominal distension, vomiting, acid regurgitation, *etc.* Modern research demonstrates that DHE is one of the main components of EF. In recent years, DHE has received extensive attention due to its various pharmacological activities. This review is the first to comprehensively summarize the current studies on pharmacokinetics profiles, pharmacological properties, and toxicological risks of DHE in diverse diseases. Pharmacokinetic studies have shown that DHE has a relatively good oral absorption effect in the mean concentration curves in rat plasma and high absorption in the gastrointestinal tract. In addition, distribution re-absorption and enterohepatic circulation may lead to multiple blood concentration peaks of DHE in rat plasma. DHE possesses a wide spectrum of pharmacological properties in the central nervous system, cardiovascular system, and digestive system. Moreover, DHE has anti-inflammatory effects *via* downregulating pro-inflammatory cytokines and inflammatory mediators. Given the favorable pharmacological activity, DHE is expected to be a potential drug candidate for the treatment of Alzheimer’s disease, chronic stress, amnesia, chronic atrophic gastritis, gastric ulcers, and rheumatoid arthritis. In addition, toxicity studies have suggested that DHE has proarrhythmic effects and can impair bile acid homeostasis without causing hepatotoxicity. However, further rigorous and well-designed studies are needed to elucidate the pharmacokinetics, pharmacological effects, potential biological mechanisms, and toxicity of DHE.

## Introduction

**FIGURE 1:**
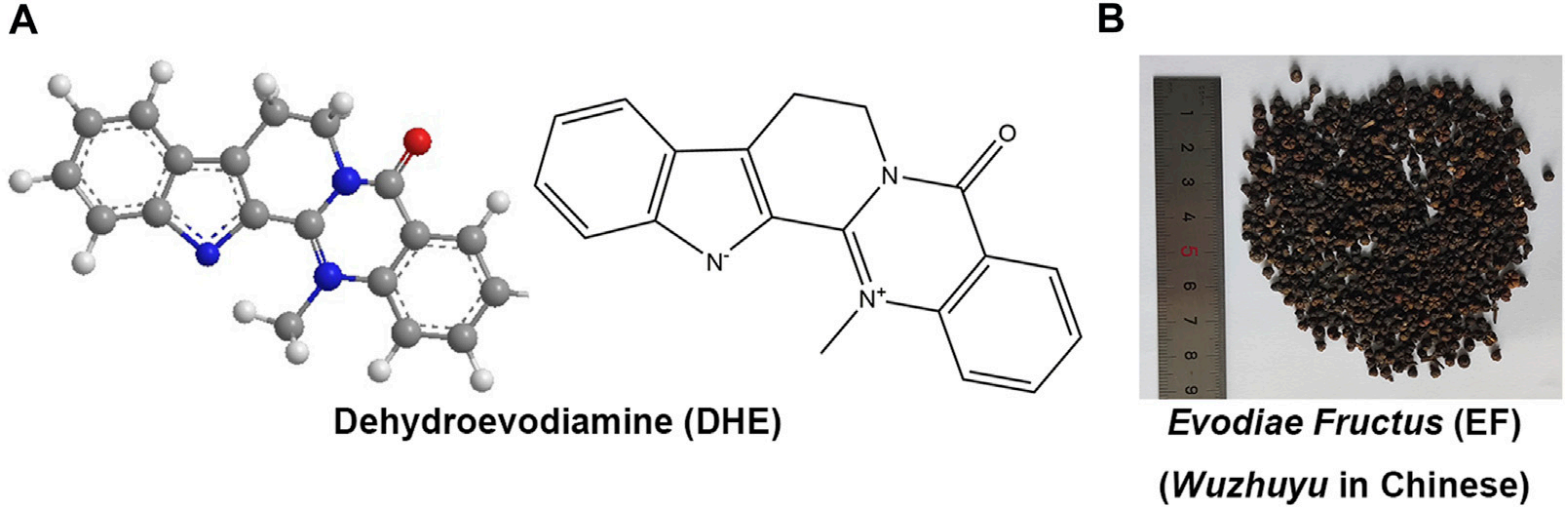
The chemical structures of DHE (PubChem CID: 9817839) **(A)** and the prepared slices of EF **(B)**. Notes: DHE, dehydroevodiamine; EF, *Evodiae Fructus*.

Dehydroevodiamine (DHE, 14-Methyl-5-oxo-7,8-dihydro-5H-indolo[2′,3':3,4]pyrido[2,1-b]quinazolin-14-ium-13-ide, Figure 1A), molecular formula: C_19_H_15_N_3_O, PubChem CID: 9817839, CAS NO. 67,909–49–3, relative molecular mass: 301.3 (https://pubchem.ncbi.nlm.nih.gov/compound/9817839), is one of the natural bioactive components derived from a widely used traditional Chinese medicine (TCM), *Evodiae Fructus* (EF, *Wuzhuyu* in Chinese, *Rutaceae* family) (Figure 1B) (Park et al., 1996; Nam et al., 2016; Wang et al., 2021). For thousands of years, EF has been widely used as a central agent in classical Chinese herbal prescriptions (*Wuzhuyu* decoction and *Zuojin* formula) to treat migraine (known as “*Jueyin* headache” in ancient China) as well as other diseases (Sun et al., 2020). Currently, EF is still a commonly used drug in China for the treatment of various diseases, such as headaches, oral ulcers, menstrual discomfort, cardiovascular diseases (CVD), gastrointestinal diseases, and central nervous system diseases (Schramm and Hamburger, 2014; Tian et al., 2019). Alkaloids are traditionally considered to be the primary biologically active compounds in EF, not only because of the isolation of various types of alkaloids from herbal medicines but also because pharmacological and clinical studies have shown that the main chemical constituents in EF are alkaloids (Han et al., 2007; Gong et al., 2009). EF mainly contains chemical components such as indoloquinazoline, quinolone, limonoid alkaloids, and flavonoids (Sugimoto et al., 1998; Huang et al., 2012; Wang et al., 2013).

DHE is a white crystalline powder, which is usually soluble in methanol, ethanol, dimethyl sulfoxide (DMSO), and other organic solvents. Evodiamine (PubChem CID: 442088), rutaecarpine (PubChem CID: 65752), and DHE are the three main biological components derived from EF. All three have the same basic structure as indoloquinazoline alkaloids, with different substituents only at the N-14 atom, which might be the critical factor affecting the three-dimensional conformation change and pharmacological activities of DHE compared with the other two alkaloids (Perkins et al., 2014). In general, DHE is not commercially available. Schramm et al., devised a simple, robust, and scalable procedure to purify DHE at the Gram scale. Simultaneously, the process for the selective removal of DHE from EF extracts was also designed to deal with the drug development and clinical practice of DHE (Schramm and Hamburger, 2014).

Quantitative analysis by liquid chromatography-mass spectrometry (LC-MS) showed that the chloroform extract of 1 g of EF decoction contained 0.60 mg of DHE (Ueng et al., 2002). Due to higher polarity, DHE is the active compound in a higher yield in EF. Chromatographic separations revealed a limit of quantifications of 6.88 ng/ml for DHE in EF (Zhao et al., 2014). Modern researches confirm that DHE exerts extensive pharmacological activities *in vitro* and *in vivo*, such as anti-inflammatory, anti-hypertensive, anti-cancer, anti-thrombotic, anti-obesity, anti-cholinesterase, anti-amnestic, analgesic, neuroprotective, and vasodilatory activities (Yang et al., 1990; Wang et al., 2001; Lim et al., 2004; Zhang Y. N. et al., 2018; Tian et al., 2019; Dai et al., 2022). Thus, the potential therapeutic effects of DHE have been shown on treating Alzheimer’s disease (AD), chronic stress, amnesia, chronic atrophic gastritis (CAG), gastric ulcers, and rheumatoid arthritis (RA) (Loh et al., 2014; Schramm and Hamburger, 2014; Ahmad et al., 2021; Wen et al., 2021; Dai et al., 2022). Based on its broad effectiveness, DHE has attracted much attention as a promising chemical compound and natural product for the prevention and treatment of diverse diseases.

The pharmacokinetics profiles, pharmacological properties, toxicological risks, and biological mechanisms of DHE have been extensively reported in the past decades. However, most of the previous related studies are scattered reports, lacking a systematic and comprehensive summary and induction of DHE. Therefore, this review aims to provide a comprehensive summary and discussion of the latest research progress on the pharmacokinetics profiles, pharmacological properties, and toxicological risks of DHE in various diseases, which will be beneficial to the further clinical practice and application of DHE.

## Materials and methods

### Source material

DHE, a quinazoline alkaloid derived from *Evodiae Fructus* (EF, *Wuzhuyu* in Chinese, Rutaceae family), which is the dry near-ripe fruit of *Evodia rutaecarpa* (Juss.) Benth., *Evodia rutaecarpa* (Juss.) Benth. var. o*fficinalis* (Dode) Huang or *Evodia rutaecarpa* (Juss.) Benth. var. *bodinieri* (Dode) Huang (Nam et al., 2016; Wang et al., 2021). EF belongs to Rutaceae family, *Tetradium ruticarpum* (A. Juss.) T. G. Hartley [Rutaceae] (http://mpns.kew.org/mpns-portal/; http://www.plantsoftheworldonline.org). The non-scientific name, class of name, and medicinal source of EF are shown in Supplementary Table S1.

### Methods

The information of this review was comprehensively searched with resources from multiple literature databases, including PubMed, EMBASE, Web of Science, Wiley Online Library, SinoMed, China National Knowledge Infrastructure (CNKI), VIP medicine information system (VMIS), Wanfang, Chinese Biomedical Database (CBM) and so on. The following search terms were used, including “dehydroevodiamine”, “pharmacokinetics”, “pharmacology”, “toxicology” and so on. Studies concerning the role of DHE in pharmacokinetics profiles, pharmacological properties, and toxicological risks in various diseases were picked out manually. The related studies were downloaded for further evaluation.

### Ethics approval and consent to participate

As this study does not involve animal and patient experiments, the ethics approval and consent to participate are not applicable.

### Pharmacokinetics profiles of DHE

Studies have investigated the pharmacokinetics of DHE in rats, mice, as well as *in silicon* in recent years. Ahn et al., investigated the distribution kinetics of DHE in the rat brain (Ahn et al., 2004). The time profile of DHE plasma levels decreased in a multi-exponential manner after 15 min of intravenous infusion (at doses of 1–10 mg/kg). Moment analysis indicated that the pharmacokinetics of DHE was linear over the range examined. The concentrations of DHE in cerebrospinal fluid were negligible compared to those in plasma, indicating that the drug is not primarily distributed to the brain through the blood-cerebrospinal fluid barrier. The kinetic analysis indicates that the distribution of DHE inside and outside the brain is regulated by first-order kinetic (Table 1). Xu et al., detected the non-compartmental pharmacokinetic parameters of eight chemical components including DHE in rat plasma (Xu et al., 2013). After oral administration, the plasma concentration of DHE increased rapidly, reaching C_max_ within 1 h, and then decreased sharply in a short period of time. Moreover, the results showed that the pharmacokinetic parameters of DHE were statistically different between normal and headache rats (Table 1). The quaternary ammonium structure of DHE might serve as a suitable substrate for organic cation transporters expressed in the rat gut (Zhang et al., 1998). Therefore, DHE might be actively secreted in the gut, leading to irregular absorption patterns.

**TABLE 1:** Pharmacokinetic parameters of DHE.

Species	Dose of DHE	Pharmacokinetics parameters
Rats Ahn et al. (2004)	Intravenous infusion of 15 min (1–10 mg kg^−1^)	*Moment Analysis* (*2.5 mg kg* ^*−1*^ *)*
		*AUC*_*0→∞*_: 52,500 ± 19,400 ng min·mL^−1^
		*AUMC*_*0→∞*_: 10,800,000 ± 3,980,000 ng min^2^·mL^−1^
		*MRT*_*apparent*_: 197 ± 6.72 min
		*CL*: 53.7 ± 25.0 ml min^−1^·kg^−1^
		*V*_*ss*_: 10,200 ± 4680 ml kg^−1^

		*Simultaneous Nonlinear Regression Analysis* (*2.5 mg kg* ^*−1*^ *)*
		*C*_*1*_: 2000 ± 1790 ng mL^−1^
		*C*_*2*_: 110 ± 6.94 ng mL^−1^
		*λ*_*1*_: 0.729 ± 0.213 min^−1^
		*λ*_*2*_: 0.00585 ± 0.000223 min^−1^
		*CL*_*influx*_: 0.0373 ± 0.0191 ml min^−1^
		*CL*_*efflux*_: 0.0431 ± 0.0181 ml min^−1^
Rats Xu et al. (2013)	Intragastric administration with 6.67 g kg^−1^ *Wuzhuyu* decoction	*Normal Group*
		*AUC*_*0→t*_: 635.4 ± 282.0 ng h·mL^−1^
		*AUC*_*0→∞*_: 689.1 ± 268.7 ng h·mL^−1^
		*C*_max_: 432.4 ± 258.9 ng mL^−1^
		*T*_*1/2*_: 3.81 ± 2.02 h
		*T*_max_: 0.77 ± 0.00 h

		*Headache Group*
		*AUC*_*0→t*_: 720.9 ± 220.5 ng h·mL^−1^
		*AUC*_*0→∞*_: 740.9 ± 216.6 ng h·mL^−1^
		*C*_max_: 564.0 ± 246.2 ng mL^−1^
		*T*_*1/2*_: 8.79 ± 3.27 h
		*T*_max_: 0.77 ± 0.00 h
C57BL/6N mice Zhang et al. (2018b)	80 mg/kg DHE by gavage	*LogP*: 2.30
		Permeability (cm·s^−1^) (pH = 7.4): 2.00 × 10^−7^
		Intrinsic solubility (mg·ml^−1^) (pH = 7.4): 0.14
		Topological polar surface area (Ǻ^2^): 39.1
		*T*_*1/2*_: 64.0 ± 3.45 min
		*T*_max_: 60.0 ± 0.1 min
		*C*_max_: 12.8 ± 2.39 μg ml^−1^
		*AUC*_*0∼t*_: 3450 ± 34.6 μg ml^−1^·min
		*AUC*_*0∼∞*_: 4760 ± 36.1 μg ml^−1^·min
Rats Li et al. (2021)	A single oral administration of an extract mixture of 2 g/kg *Tetradium ruticarpum* fruit and 2 g/kg licorice	*C*_max_: 8.25 ± 2.48 ng mL^−1^
		*T*_max_: 3.77 ± 2.89 h
		*T*_*1/2*_: 2.35 ± 1.27 h
		*AUC*_*0-t*_: 49.65 ± 12.60 ng h·mL^−1^
		*AUC*_*0-C*_: 53.19 ± 12.50 ng h·mL^−1^
		MRT: 5.78 ± 0.93 h
		*k*: 0.34 ± 0.10 h^−1^
Rats Qian et al. (2017)	Orally administered with 12 g/kg *Zuojin formula* (crude drug dose)	*AUC*_*0→t*_: 532.34 ± 57.78 ng h·mL^−1^
		*AUC*_*0→∞*_: 537.43 ± 54.97 ng h·mL^−1^
		*MRT*_*0→t*_: 260.43 ± 15.40 min
		*MRT*_*0→∞*_: 282.90 ± 34.14 min
		*T*_*1/2*_: 192.57 ± 135.94 min
		*T*_max_: 240 min
		*C*_max_: 155.16 ± 27.92 ng mL^−1^
		*T*_*sec*_: 90 min
		*C*_*sec*_: 117.29 ± 45.45 ng mL^−1^
	Orally administered with 12 g/kg *Fan-Zuojin* formula (crude drug dose)	*AUC*_*0→t*_: 274.77 ± 23.19 ng h·mL^−1^
		*AUC*_*0→∞*_: 285.60 ± 24.08 ng h·mL^−1^
		*MRT*_*0→t*_: 279.59 ± 56.33 min
		*MRT*_*0→∞*_: 357.86 ± 114.71 min
		*T*_*1/2*_: 301.96 ± 172.11 min
		*T*_max_: 90 min
		*C*_max_: 85.27 ± 13.37 ng mL^−1^
Rats Yan et al. (2012)	Oral gavage with 0.18 g EF and *Zuojin* formula powder/kg body weight	EF
		*AUC*_*0→t*_: 68,130 ± 17,451 μgH·L^−1^·h^−1^
		*AUC*_*0→∞*_: 68,134 ± 19,162 μgH·L^−1^·h^−1^
		*MRT*_*0→t*_: 4.6 ± 0.7 h
		*MRT*_*0→∞*_: 4.6 ± 5.6 h *t*_*1/2z*_: 1.6 ± 6.4 h
		*T*_max_: 3.5 ± 3.0 h
		*C*_max_: 15,383 ± 7,166 μgH·L^−1^
		Zuojin Formula
		*AUC*_*0→t*_: 186,698 ± 46,442 μgH·L^−1^·h^−1^
		*AUC*_*0→∞*_: 186,715 ± 39,211 μgH·L^−1^·h^−1^
		*MRT*_*0→t*_: 4.9 ± 0.7 h
		*MRT*_*0→∞*_: 4.9 ± 3.6 h *t*_*1/2z*_: 1.8 ± 5.4 h
		*T*_max_: 1.5 ± 1.1 h
		*C*_max_: 40,992 ± 21,052 μgH·L^−1^
*In silicon* Ahmad et al. (2021)	Not report	With high gastrointestinal absorption
		Log *Kp* (skin permeation): 6.64 cm s^−1^
		Bioavailability Score: 0.55

Notes: EF, evodiae fructus; AUC, area under concentration-time curve; MRT, mean residence time; CL, clearance.

Intestinal solubility is a crucial factor affecting the absorption of various components. Zhang et al., calculated the pharmacokinetic parameters of DHE and predicted the physicochemical parameters *in silico* (Zhang Y. et al., 2018). The computer analysis of physicochemical predictions indicated an intrinsic solubility of 0.14 mg/ml for DHE. Male C57BL/6N mice were given DHE at a dose of 80 mg/kg by gavage for 21 days. Calculation of pharmacokinetic parameters showed that the C_max_ values of DHE treatment were 12.8 ± 2.39 μg/ml. Ahmad et al., selected the most active natural compounds from a library of 224, 205 compounds in the ZINC database (Ahmad et al., 2021). According to the pharmacokinetic analysis, DHE has a higher binding free energy to the acetylcholinesterase (AChE) receptor. The energy information obtained by the docking between DHE and AChE is as follows: binding energy: 9.00 kcal/mol; inhibition constant: 4.25 μM; intermolecular energy: 7.50; Van der Waals’, ‘Hydrogen Bond’ and ‘Desolvation Energy’: 7.46; electrostatic energy: 0.05.

Previous studies have shown that DHE could be used as a representative component reflecting the pharmacokinetic behavior of EF alkaloids after administration of the *Zuojin* formula (*Coptidis Rhizoma*: EF = 6: 1) and *Fan-Zuojin* formula (*Coptidis Rhizoma*: EF = 1: 6). Li et al., analyzed the pharmacokinetic profiles of DHE by developed LC-MS/MS using rat plasma after a single oral administration of an extract mixture of 2 g/kg *Tetradium ruticarpum* fruit (*Wuzhuyu* in Chinese) and 2 g/kg licorice (the root and rhizome of *Glycyrrhiza uralensis* Fisch., *Glycyrrhiza glabra L.,* and *Glycyrrhiza inflata* Batal., *Glycyrrhiza uralensis* Fisch., *Gancao* in Chinese) (Li et al., 2021). The pharmacokinetic parameters of DHE show that the maximum plasma drug concentration is 8.25 ng/ml. The other pharmacokinetic values of DHE are shown in Table 1. Qian et al., performed a comprehensive pharmacokinetic study to compare the pharmacokinetic parameters between the *Zuojin* formula and the *Fan-Zuojin* formula (both are mainly composed of *Coptidis Rhizoma* and EF to illustrate the compatibility dose effect) (Qian et al., 2017). Finally, the pharmacokinetic profiles of 12 alkaloids including DHE after oral administration of the *Zuojin* formula and *Fan-Zuojin* formula were compared *in silico*. The results showed that compared with evodiamine, DHE had a higher level of systemic exposure, regardless of the dose. The C-T curve of DHE is most similar to the comprehensive C-T curve of EF alkaloids, suggesting that DHE can be considered as a representative component reflecting the pharmacokinetic behavior of EF alkaloids. After oral administration of *Wuzhuyu* decoction (6.67 g kg^−1^) to rats in the control group and headache group. Yan et al., used a rapid LC-MS method to evaluate the comparative pharmacokinetic parameters of DHE in rats’ plasma after oral administration of EF and *Coptidis Rhizoma*-EF powders (*Zuojin* formula) combination (Yan et al., 2012). The stability of low and high concentrations of DHE in rat plasma has been comprehensively evaluated under various storage and processing conditions. The stability studies have shown that DHE is stable in plasma for 4 h at room temperature (25°C), after three freeze-thaw cycles, and after the reconstitution at 25°C for 24 h. Taken together, DHE has higher exposure and is well absorbed *in vivo*. The pharmacokinetic parameters of DHE are summarized in Table 1.

## Pharmacological effects of DHE

### Effects of DHE on the central nervous system

DHE has a clear protective effect on the central nervous system. In recent years, a large number of literatures have reported that DHE has a preventive effect on AD induced by various models, and it exhibits good blood-brain barrier (BBB) permeability. DHE is the main component of EF for neuroprotective effect, and it has a neuroprotective effect on the PC12 cell line damaged by MPP^+^ or H_2_O_2_ (Zhang Y. N. et al., 2018).

AD is a typical and fatal neurodegenerative condition with no available preventive treatments (Kandimalla and Reddy, 2017; Ahmad et al., 2019). Currently, cholinesterase inhibitors (ChEIs) are the treatment of choice for AD based on clinical studies on the effects of drugs on cognition (memory and attention) and behavioral symptoms (apathy and agitation) (Decker, 2005). Also, ChEIs have been officially approved clinically for the symptomatic treatment of AD (Han et al., 2019). Numerous studies claim that DHE has substantially pharmacological effects of anti-AChE and enhances cognitive function in memory-impaired rat models, and thus has the effect of treating AD. DHE exhibits strong anti-amnestic activity *in vivo* and moderate AChE inhibition *in vitro* (Table 2) (Park et al., 1996). Its efficacy is due in part to AChE inhibition, but also the long-term promotion of synaptic transmission due to the activation of muscarinic and N-methyl-D-aspartate receptors (Park et al., 2003). Lim et al., found that DHE inhibited the uptake and release of glutamate, suggesting that chronic exposure to DHE might alter the characteristics of glutamate release and uptake in granule and glial cells (Lim et al., 2004).

**TABLE 2:** Pharmacological effects of DHE on various central nervous system diseases.

Pharmacological activities	Dose and route of administration	The experimental model of diseases	Mechanisms	References
Restore cognitive and memory deficits				
Reduce AD pathological damage associated with Tau	1 mg/kg, *i.p*	The 5xFAD Tg AD mouse model	Synaptic-related proteins, such as GluN2A-containing NMDARs and PSD-95, Tau protein	Kang et al. (2018)
Restore memory and cognitive impairment				
Anti-oxidation				
Inhibit neurotoxicity				
Decrease intracellular calcium levels	10 mg/kg, *p.o*	Scopolamine-induced amnesia and a Aβ_1-42_-infused model	ROS, Aβ_1-42_ peptide, neurotoxicity, intracellular calcium levels	Shin et al. (2017)
Restore memory and cognitive impairment				
Antagonize Aβ deposition	0.5 mg/kg, *i.p*	APP695 transgenic mice	Aβ40, Aβ42, β-secretase	Shin et al. (2016)
Improve memory impairments and depression-like behaviors	10 mg/kg, *p.o*	Immobilization-induced chronic stress in rats	NCAM proteins	Kim et al. (2014)
Anti-oxidative stress	3, 6 mg/kg, *p.o*	D-galactose-induced subacute aging model	SOD	Kang et al. (2010)
Inhibit Tau protein hyperphosphorylation				
Alleviate spatial memory deficit	6.25, 12.5 mg/kg, Tail vein injection	WT/GFX-induced tau hyperphosphorylation and memory impairment rats	GSK-3, Tau protein	Peng et al. (2007)
Decrease inhibitory phosphorylation of PP-2A at Tyr307	10, 100, 200 μM,pre-incubated at 33 °C	Calyculin A-induced AD-like tau hyperphosphorylation	Tyr307-phosphorylated PP-2A, Tau protein	Fang et al. (2007)
Anti-cholinesterase and improve Aβ type amnesia	0.3–12 mg/kg, *i.p*	Scopolamine-and Aβ peptide-(25–35)-induced amnesia in mice	Aβ peptide-(25–35)	Wang et al. (2001)
Improve impaired spatial working memory				
Improve cognitive deficits				
Reduce neuron loss and infarct size	6.25 mg/kg, *i.p*	Scopolamine-induced amnesia model of the rat, the MCA-occluded model, and the electrolytic lesioned model of the entorhinal cortex	Protective effects on cognitive deficits and neuronal loss	Park et al. (2000)
Anti-cholinesterase and anti-amnesic	6.25 mg/kg, *i.p*	Scopolamine-induced amnesia model in rats	Anti-AChE activity	Park et al. (1996)

Notes: AD, Alzheimer’s disease; p. o., peros; i. p., intraperitoneal injection; ROS, reactive oxygen species; SOD, superoxide dismutase; AChE, acetylcholinesterase; GSK-3, glycogen synthase kinase-3; Aβ, β-amyloid; WT/GFX, wortmannin and GF-109203X; tg, transgenic; NMDARs, N-methyl-D-aspartate receptors; NCAM, neural cell adhesion molecule; MCA, middle cerebral artery.

It has been reported that from the comprehensive effect of cerebral blood flow enhancement and AChE inhibition, the natural product-based DHE is less effective on AChE than tacrine, but its anti-amnestic effect is more effective than that of tacrine (Park et al., 1996). The anti-AChE activity of DHE has an IC_50_ value of 37.9 μM in the treatment of AD, which could be used as a positive anti-AD drug in experimental studies (Zhang et al., 2013). Also, Jung et al., reported that the IC_50_ of DHE hydrochloride for inhibitory AChE activity was 37.8 μM (Jung and Park, 2007). Ahmad et al., concentrated on screening natural compounds capable of managing AChE from the ZINC database (224, 205 compounds) (Ahmad et al., 2021). The results indicated that DHE was one of the most potential AChE inhibitors with a free binding energy of -9.00 kcal/mol. Moreover, DHE might cross the BBB and exhibit high levels of intestinal absorption. However, researchers still need to design more experimental studies for using DHE in the treatment of AD.

Changes in compound structure always lead to changes in pharmacokinetics and pharmacological effects. Carboxy-dehydroevodiamine·HCl (cx-DHED) is a derivative of DHED, which increases its solubility in water, enhances its high bioavailability, and is superior to DHE in ameliorating memory impairment. The study by Kang et al., showed that cx-DHED has a clear therapeutic effect on 5xFAD and AD model mice by improving synaptic stability, which could dramatically reduce memory impairment, amyloid plaque numbers, and PHFs-tau, as well as synaptic instability in 5xFAD AD mice (Kang et al., 2018). Thus, these results suggested that cx-DHED could prevent the development and progression of AD pathology as well as memory deficits in 5xFAD mice (Table 2). Also, Kim et al., found that DHEHCl could prevent memory impairment and neuronal cell loss in a rat model of cognitive impairment (Kim et al., 2014). The effects of DHEHCl on stress-induced memory impairment and behavioral abnormalities were investigated. Mechanistic studies showed that DHEHCl treatment significantly restored the stress-induced reduction in neural cell adhesion molecule (NCAM) protein levels as well as cell viability (Table 2). The results suggested that DHEHCl is a potential drug candidate for memory impairment, neuronal death, and stress-induced depression.

In addition, Shin et al., investigated the effects and potential mechanism of DHE on cognitive improving effect in a scopolamine-induced amnesia model and an Aβ_1-42_-infused memory-impaired rat model (Shin et al., 2017). The findings suggest that 10 mg/kg DHE (*p.o.*) has a strong protective effect against cognitive impairment via its anti-oxidant activity, such as a downregulation in ROS production, and inhibition of neurotoxicity as well as intracellular calcium levels. DHE might be a useful therapeutic agent for symptoms of memory impairment such as AD (Table 2). Fang et al., investigated the effect of DHE on the protein phosphatase (PP)-2A and the PP-1 inhibitor calyculin A (CA)-induced AD-like tau hyperphosphorylation, and its involvement in PP-2A content in metabolically competent rat brain slices (Table 2) (Fang et al., 2007). Rat brain sections were pre-incubated for 1 h at 33 °C in the presence or absence of DHE (10, 100, and 200 μM, respectively). Then CA 0.1 μM was added and treated for another 2 h. DHE was found to relieve CA-induced tau hyperphosphorylation at multiple AD-associated sites in metabolically active rat brain slices. The underlying mechanism may involve decreased inhibitory phosphorylation of PP-2 A at Tyr307. Wang et al., examined the protective effects of DHE (0.75–12.0 mg/kg, *i. p.*) on scopolamine-as well as β-amyloid (Aβ) peptide-(25–35)-induced amnesia in mice via a step-through passive avoidance test (Table 2) (Wang et al., 2001). It was found that DHE was more effective in amnesia-induced amnesia than Aβ peptide-(25–35)-induced amnesia. DHE has the effect of anti-cholinesterase, and may also be a new effective ligand for improving Aβ amnesia.

Since DHE readily crosses the BBB, it has been reported to have minimal side effects and doses among cholinesterase inhibitors (Park et al., 1996; Park et al., 2000; Ahn et al., 2004), and in addition to the current findings, it might be a promising candidate for the drug development of AD. Park et al., investigated the pharmacological effects of DHE on a scopolamine-induced amnesia model in rats (Park et al., 2000). A single (20 mg/kg, *p. o.*) and repeated (10 mg/kg, *p. o.*) dosing of DHE significantly reversed the latency of scopolamine (1 mg/kg, *i. p.*) to control levels. Furthermore, DHE dramatically improved the impaired spatial working memory and cognitive deficits in rats, as well as reduced neuronal loss and infarct size (Table 2). These results suggest that DHE might be an effective drug not only for AD types but also for vascular dementia and stroke. Also, Park et al., screened natural products with anti-amnestic activity for their ability to inhibit AChE and reverse scopolamine-induced amnesia. DHE was found to strongly inhibit AChE activity in a dose-dependent and non-competitive manner *in vitro* and exhibited anti-amnestic effects *in vivo*. DHE has an IC_50_ value of 37.8 μM. This potent anti-amnestic effect of DHE is thought to be due to the combined effect of AChE inhibition and the known enhancement of cerebral blood flow. DHE increased cerebral blood flow recorded from the surface of the lateral gyrus of the brain in anesthetized cats. This action reached a maximum 1–4 min after injection and continued for 10 min. However, at the doses examined, the compound had negligible effects on other cardiorespiratory functions (Table 2). These results suggest that DHE selectively increases cerebral blood flow (Haji et al., 1994). Kang et al., discussed the effect of DHE on the learning and memory ability and anti-oxidant capacity of D-galactose-induced aging model mice, and preliminarily discussed the mechanism of action (Kang et al., 2010). The experimental results show that DHE could improve the learning and memory dysfunction of the aging mouse, which might play an anti-oxidative stress role by regulating the level of superoxide dismutase (SOD) (Table 2).

### Effects of DHE on the cardiovascular system

DHE has clear cardiovascular pharmacological activity, which has an anti-arrhythmic effect on guinea pig ventricular myocytes. Yang et al., investigated the cardiovascular effects of DHE *in vivo* and *in vitro* (Yang et al., 1990). The intravenous injection administration of DHE caused a slight decrease in blood pressure *in vivo*, a significant decrease in heart rate, and an increase in ECG cycle length. However, DHE did not alter total peripheral resistance. Except for reduced blood flow to the kidneys and skin, there were no significant changes in blood flow to other organs. Moreover, DHE significantly inhibited spontaneously beating atria in a dose-dependent manner *in vitro*. These findings suggest an important role for DHE in suppressing the heart, which may largely contribute to the anti-hypertensive effects of this alkaloid. However, its vasodilatory effect on hindquarters muscles cannot be ignored.

DHE has been reported to have vasodilatory effects (Chiou et al., 1996b). It could induce vasodilatory effects on rat aorta with intact endothelium through partial endothelium-dependent effects, R1-adrenoceptor blockade, and 5-hydroxytryptamine (5-HT) antagonism (Chiou et al., 1996a). Electrophysiological studies on isolated guinea pig cardiomyocytes indicated that DHE inhibited cardiac ion currents of *I*_*Na*_ *, I*_*Ca,L*_ *,* and *I*_*K*_. Simultaneously, it prolonged the duration of action potentials in the ventricle and atrium of the guinea pig (Yang et al., 1990).

DHE reduces arterial blood pressure and prolongs the duration of action potentials in cardiomyocytes in experimental animals. Loh et al., explored the ionic basis of its possible anti-arrhythmic effects (Loh et al., 1992). Studies have shown that DHE might suppress arrhythmias triggered in Ca-overloaded guinea pig cardiomyocytes through its inhibitory effects on *I*_*Na*_, *I*_*ti*_, and to a lesser extent *I*_*Ca*_. DHE also exerts class III anti-arrhythmic effects by reducing the outward K current (*I*_*k*_) across the sarcolemma. DHE (0.1–0.3 μM) is highly effective in inhibiting cardiac arrhythmias induced in Ca^2+^-overloaded guinea-pig cardiomyocytes in low K^+^ and high Ca^2+^ perfusates in guinea-pig cardiomyocytes. The ionic underlying mechanism of the cardioprotective effect of DHE is mainly due to its inhibitory effect on *I*_*Na*_ and *I*_*Ca*_ (Loh et al., 1992). Loh et al., investigated the electromechanical effects of DHE in human atrial and ventricular tissue (Loh et al., 2014). In human atrial and ventricular myocardium, DHE (0.1–0.3 μM) reduced slow- and fast-response action potential ascending velocity, action potential amplitude, and contractility. DHE (0.1–1 μM) reversibly and concentration-dependently decreased Na^+^ and Ca^2+^ currents in isolated human atrial and ventricular myocytes. In the human ventricular myocardium, strophanthidin-induced triggering activity was attenuated by pretreatment with DHE (0.3 μM). In addition, DHE (0.1–0.3 μM) also memorably increased resting pHi and Na^+^-H^+^ exchanger (NHE) activity. In the human heart, DHE could antagonize inotrope-induced arrhythmias by generally reducing Na^+^ and Ca^2+^ inward currents while increasing resting intracellular pH (pHi) and NHE activity.

DHE has been reported to induce bradycardia in anesthetized rats (Xu et al., 1982; Yang et al., 1990). It inhibits aortic constriction *in vitro* as a calcium antagonist and has been suggested to have calcium-blocking activity on calcium currents in the mammalian heart (Loh et al., 1992). Wong investigated whether DHE could act as a calcium antagonist on chronotropic and inotropic activity in isolated mouse atria (Wong, 1996). The data showed that DHE induced bradycardia, but did not reduce right atrial contraction amplitude. Furthermore, DHE did not attenuate the amplitude of contraction of the electrically driven left atrium, and in the presence of 1 × 10^−4^ M DHE, the amplitude of contraction of the left atrium increased when the calcium concentration in Krebs solution was further increased. Since calcium antagonists are known to inhibit chronotropic and inotropic activity, it seems unlikely that DHE acts as a calcium antagonist in chronotropic and inotropic activity in isolated mouse atria.

### Effects of DHE on the digestive system

In addition to the effects of DHE on cardiovascular and central nervous system cognition, it also has certain pharmacological effects on the digestive system. Wei et al., clarified the pharmacological effects and mechanisms of DHE on indomethacin (IDO)-induced gastric injury (Wei et al., 2021). The study found that DHE attenuated IDO-induced decreased food intake, weight loss, and gastric injury, and normalized gastric pH and mucosal thickness. In addition, DHE down-regulates the expression of myeloperoxidase (MPO), tumor necrosis factor-α (TNF-α), and interleukin-6 (IL-6), and up-regulates the expression of interleukin-10 (IL-10) to reduce inflammation-induced damage and create a healing environment. Furthermore, DHE could significantly inhibit the phosphorylation of extracellular signal-regulated kinase (ERK) and p38 but not c-Jun N-terminal kinase (JNK). Studies have shown that DHE ameliorated IDO-induced dyspepsia, inflammatory infiltration, and tissue damage through the ERK and p38 signaling pathways but not the JNK pathway (Table 3). Wen et al., established a rat CAG model and a GES-1 human gastric epithelial cell injury model using N-methyl-N′-nitro-N-nitrosoguanidine (MNNG) and investigated the therapeutic effect and potential molecular biological mechanism of DHE on CAG (Wen et al., 2021). The results showed that the therapeutic effect of DHE on CAG rats was manifested by down-regulating the levels of serum inflammatory factors and reducing histological damage to gastric histology. In addition, DHE was effective in increasing cell proliferation of GES-1 cells, ameliorating MNNG-induced gastric epithelial cell damage and mitochondrial dysfunction. Molecular biological mechanism studies have shown that DHE has a regulatory effect on tumor angiogenesis, and can play an anti-CAG effect by inhibiting the relative expression of genes and proteins related to the vascular endothelial growth factor (VEGF) signaling pathway mediated by hypoxia-inducible factor-1 alpha (HIF-1α) (Table 3).

**TABLE 3:** Pharmacological activities of DHE in the digestive system.

Effects (Reference)	Animals or cells	Experimental model	Doses of DHE	Pharmacological effects	Pathways
Treatment for CAG Wen et al. (2021)	Rats	170 μg/ml of MNNG-induced CAG	5, 10 mg/kg, *i.g.*	Down-regulate serum inflammatory factor levels Alleviate histological damage of gastric tissue Increase cell proliferation of GES-1 cells Ameliorate MNNG-induced gastric epithelial cell damage and mitochondrial dysfunction Inhibit migration and invasion of GES-1 cells	Regulate inflammation metabolites and energy metabolism-related pathways. Inhibit HIF-1α/VEGF angiogenesis pathway
Treatment for gastric ulcers Wan and Bao, (2020)	Rats	50 μl glacial acetic acid-induced stomach ulcer	6.25, 12.5 mg/kg, *i.g.*	Reduce gastric mucosal ulcers area, serum oxidative stress factor, and serum inflammatory factor levels Increase gastric ulcers inhibition rate, gastric mucosal repair factor levels	Regulate Rho/NF-κB signaling pathway
Treatment for gastric ulcers Wei et al. (2021)	Rats	5 mg/kg IDO-induced gastric ulcers	10, 20, 40 mg/kg, *i.g.*	Relieve gastric injury, restore gastric pH and mucosal thickness. Improves indigestion. Alleviate inflammatory infiltration and tissue damage. Create a healing environment	Regulate the ERK and p38 signaling pathways but not the JNK pathway

*Notes: CAG, chronic atrophic gastritis; MNNG, N-methyl-N’-nitro-N-nitrosoguanidine; HIF-1α, hypoxia-inducible factor-1 alpha; VEGF, vascular endothelial growth factor; NF-κb, nuclear factor kappa-B; IDO,* indomethacin.

**FIGURE 2:**
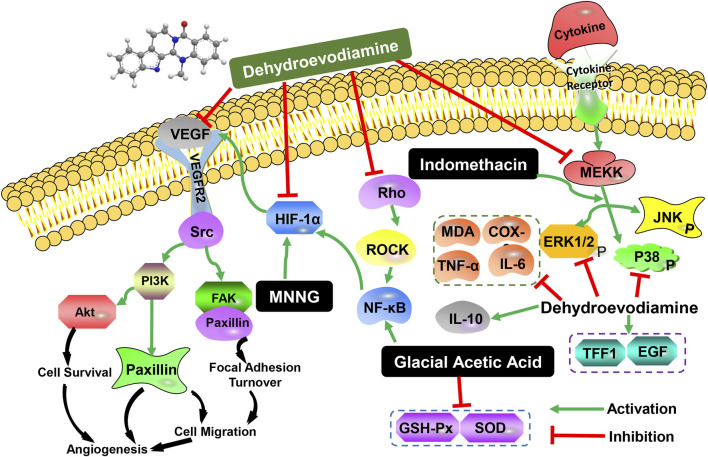
The main biological activities and possible molecular mechanisms of DHE on the digestive system.

In addition, Wan et al., discussed the protective effect and mechanism of DHE on the gastric mucosa of rats with gastric ulcers caused by acetic acid cauterization (Wan and Bao, 2020). The results indicated that DHE has an effect on inhibiting Rho/nuclear factor kappa-B (Rho/NF-κB) signaling, regulating the inflammatory response in gastric ulcer rats, alleviating oxidative stress, and then preventing gastric mucosal damage. Simultaneously, it could promote the release of local trefoil factor family 1 (TFF1) and gastric tissue EGF levels in the stomach and accelerate the repair of the gastric mucosa. It is shown that DHE can significantly improve gastric ulcers in rats through anti-oxidative stress and anti-inflammatory factors, and its potential mechanism may be related to the regulation of the Rho/NF-κB signaling pathway. Therefore, DHE can play a role in the treatment of gastric mucosal ulcers by inhibiting gastric mucosal damage and promoting gastric mucosal repair. The experimental data are provided for the clinical application of DHE against gastric ulcers (Table 3). Overall, the main biological activities and possible molecular mechanisms of DHE on the digestive system were shown in Figure 2.

### Anti-inflammatory effects of DHE

The anti-inflammatory effects of DHE have been reported previously (Choi et al., 2006), which could significantly down-regulate pro-inflammatory cytokines and inflammatory mediators. For example, Chiou et al., explored the possible anti-inflammatory effects of DHE by evaluating its effect on nitric oxide (NO) production in the mouse macrophage-like cell line RAW 264.7 (Chiou et al., 1997). The results showed that DHE (10, 50, 100 μM) inhibited interferon-alpha/lipopolysaccharide (IFN-α/LPS)-stimulated NO production in a concentration-dependent manner. However, DHE appears to inhibit NO production by interfering not only with the priming signal elicited by IFN-α but also with inducible nitric oxide synthase (iNOS) protein synthesis.

In recent years, researchers have devoted themselves to finding effective agents for the treatment of RA. It is found that numerous amounts of small molecule compounds derived from natural products have effects on enhancing the therapeutic effects of RA by abating inflammation reactions and inhibiting the abnormal proliferation of fibroblasts. Dai et al., explored the therapeutic effect and feasible mechanism of DHE on RA in complete freund’s adjuvant (CFA)-induced adjuvant-induced arthritis (AIA) *in vivo* and *in vitro* (Dai et al., 2022). The results indicated that DHE could substantially improve the symptoms of joint redness and joint swelling in AIA rats. Simultaneously, DHE could inhibit the serum level of pro-inflammatory factors, including TNF-α, interleukin-1 beta (IL-1β), IL-6, and interleukin-17 (IL-17), as well as the relative mRNA expression of matrix metallopeptidase 1 (MMP-1) and matrix metallopeptidase 3 (MMP-3) in MH7A arthritis synovial fibroblasts. DHE plays a role in the treatment of RA by reducing inflammation and inhibiting the abnormal proliferation of fibroblasts. Moreover, the potential mechanism might be related to the regulation of the mitogen-activated protein kinase (MAPK) pathway.

### Toxicological risks of DHE

Toxicity and safety should be considered first when evaluating the pharmacological effects of drugs. One of the four classic and the earliest book of TCM *“Shennong’s Classic of Materia Medica”* records that EF has mild toxicity in humans and is a relatively safe herbal medicine. For EF, which mainly contains bioactive indoloquinazoline alkaloids, it is necessary to pay attention to its sensitivity to cardiac and liver safety. Although ingestion of EF decoction may induce human hepatic cytochrome P450 family one subfamily A member 1 (CYP1A), DHE does not affect 7-ethoxyresorufin O-deethylation activity (Ueng et al., 2002). The potential toxicological risks of DHE in various diseases are listed as follows according to the previous studies.

### DHE has proarrhythmic effects

The quinazoline alkaloid DHE is a potent human ether-a-go-go related gene (hERG) inhibitor in EF extract with an IC_50_ value of 253.2 ± 26.3 nM detected in patch clamp experiments. The hERG channel blocking property of EF decoction is proportional to the content of DHE (Hamburger, 2019). DHE could cause arrhythmias in chronic atrioventricular block dogs (0.33–0.5 mg/5 min) as well as anesthetized rabbits. In 8 chronic atrioventricular block dogs, DHE (0.33 mg/kg/5 min) could increase QT duration by 48 ± 10% and caused Torsade de Pointes (TdP) in 2/4 of these dogs. It is noteworthy that higher doses of DHE did not induce TdP. As for rabbits, DHE significantly increased the QT interval by 12 ± 10% (0.05 mg kg^−1^.5 min^−1^) and 60 ± 26% (0.5 mg kg^−1^.5 min^−1^) in eight rabbits, and induced TdP arrhythmia(0.5 mg kg^−1^.5 min^−1^) in two rabbits. In addition, it could concentration-dependent prolong action potential duration in dog ventricular cardiomyocytes. Early after depolarizations (EADs) were seen in 14, 67, 100, and 67% of dog ventricular cardiomyocytes after 0.01, 0.1, 1, and 10 μM of DHE (Baburin et al., 2018). Therefore, the dose-dependent pro-arrhythmic effect of DHE should raise awareness of the pro-arrhythmic effects of the widely used EF extract.

### DHE has no hepatotoxic

Besides, DHE has been reported to be potentially hepatotoxic. Zhang et al., investigated the possibility of hepatotoxicity induced by DHE (Zhang Y. et al., 2018). The livers and serum indices were analyzed in C57BL/6N mice by daily gavage of 80 mg/kg DHE for 3, 12, and 21 days. The results showed that the liver/body weight ratio of the mice did not show a marked difference during the administration period, suggesting that DHE did not cause substantial hepatomegaly. In addition, 80 mg/kg DHE treatment for 21 days did not cause memorably changes in serum alanine aminotransferase (ALT) and aspartate aminotransferase (AST) levels in mice, indicating no apparent hepatotoxicity. These results were also further confirmed by histological analysis, liver/body weight ratio, and serum biochemical analysis.

### DHE impairs bile acid homeostasis

DHE was not hepatotoxic at the doses used. However, it disrupted bile acid homeostasis in an aryl hydrocarbon receptor-dependent manner. These findings suggest that the methyl group on the N-14 atom of DHE and its pharmacokinetic behavior were the main determinants of aryl hydrocarbon receptor activation, suggesting that attention should be taken to monitor its effects on bile acid metabolism in the clinical application of EF and DHE (Zhang Y. et al., 2018). Global metabolomics was employed to analyze metabolites in mouse gallbladders. DHE treatment for 21 days could significantly increase the levels of unconjugated bile acids cholic acid, ω-muricholic, taurocholic acid and taurodeoxycholic acid, while slightly increasing α-muricholic, and β-muricholic levels. In addition, DHE could regulate the induction of CYP7A1 or bile salt export pump (BSEP) through the activation of the aryl hydrocarbon receptors.

## Conclusion and perspective

Natural products refer to the chemical constituents or their metabolites in animals, plants, and microorganisms. As an important source of drug discovery, natural products have the characteristics of diverse structures and have always been a crucial source of new drug discovery (Shen, 2015; Li, 2016; Ekiert and Szopa, 2022). In recent years, drugs such as artemisinin, ephedrine, vincristine, tripterygium glycosides, and taxol have received increasing attention due to their reliable efficacy and low toxicity (Xu et al., 2020). DHE is a key quinazoline alkaloid isolated from EF and now plays an important role in diseases of the central nervous system, digestive system, cardiovascular system diseases, *etc.* However, there are few comprehensive reports on the pharmacokinetics, pharmacological effects, biological mechanisms, and toxicology risks of DHE. This review provides a comprehensive summary of the chemical properties, pharmacokinetic characteristics, pharmacological activities, biological mechanisms, and toxicity of DHE. Pharmacokinetic studies have shown that DHE has a relatively good oral absorption effect in rats. DHE has a wide spectrum of pharmacological properties in the central nervous system, digestive system, and cardiovascular system, including anti-cholinesterase activity, anti-amnesia, anti-AD, anti-arrhythmic, gastrointestinal protection, and anti-inflammatory effects. In addition, toxicity studies have suggested that DHE has proarrhythmic effects and can impair bile acid homeostasis without hepatotoxic. However, long-term and high-dose toxicity studies in animals are still lacking. This summarized information might be helpful for future research and further development of DHE.

In the aspect of pharmacokinetics profiles, a variety of developed methods are successfully applied to determine the related pharmacokinetic parameters of DHE in rats, and mice, as well as *in silicon* studies. DHE has a relatively good oral absorption effect in the mean concentration curves in rat plasma (Li et al., 2021) and high absorption in the gastrointestinal tract (Ahmad et al., 2021). Also, distributional re-absorption and enterohepatic circulation may lead to multiple blood concentration peaks of DHE in rat plasma (Yan et al., 2012). DHE may be actively secreted in the intestine, resulting in irregular absorption patterns (Xu et al., 2013). In addition, the pharmacokinetic characterization of DHE in the rat brain was also studied. The dynamic distribution of DHE in rat brains showed that the time curve of DHE plasma level decreased exponentially. The clearance rate and steady-state distribution volume were not statistically different from the dose, indicating that the pharmacokinetics of DHE was linear in the range examined. The concentration of DHE in cerebrospinal fluid was negligible compared to that in plasma, suggesting that the drug was not distributed primarily to the brain through the blood-cerebrospinal fluid barrier. This indicates that DHE is transported from systemic circulation to the brain through the BBB. The distribution of DHE into and out of the brain is mediated by first-order kinetics. Consistent with the *in vivo* data, DHE transport across MBEC4 monolayers is all mediated by first-order kinetics (Ahn et al., 2004).

At present, the world has already entered an aging society (Joe et al., 2021). For the elderly, maintaining normal cognitive function is the guarantee of high-quality life. AD is a progressive neurodegenerative disease and the main type of dementia. In the elderly population, the most common diseases that impair the cognitive function are AD and cerebrovascular disease. Both have become diseases that seriously threaten the health of the elderly (Shin et al., 2016; Shin et al., 2017). Epidemiological and pathological data show that AD and cerebrovascular disease have many overlaps in etiology and pathology, and most sporadic AD is also a cerebrovascular disease (Santos et al., 2017). The treatment of cerebrovascular disease can also delay the occurrence and development of AD. It is especially necessary to extract active ingredients from TCM for AD treatment.

According to the current studies, DHE has a good protective function on the central nervous system and can act on the occurrence and development of AD through multiple pathways. DHE binds strongly to the active site residues of AChE and follows the drug-like properties predicted *in silico*. Further experimental evaluations of DHE may eventually lead to exciting alternative AD therapies (Ahmad et al., 2021). DHE can effectively slow down the occurrence and development of AD through neuroprotective function by repairing memory and cognitive impairment, antagonizing Aβ deposition, inhibiting Tau protein hyperphosphorylation, protecting isolated neurons, inhibiting glial cell activation and inflammatory mediators release, *etc.* Its pharmacological effect on cerebrovascular disease is reflected in the protective effect on ischemic brain injury. Studies have found that DHE can exert anti-AD effects by acting on targets such as ROS, SOD, Aβ40, Aβ42, β-secretase, GSK-3, Tau protein, NCAM proteins, *etc.* Its pharmacological effects include restoring memory and cognitive impairment, antagonizing Aβ deposition, inhibiting Tau protein hyperphosphorylation, restoring cognitive and memory deficits, improving spatial memory impairment, anti-oxidation, inhibiting neurotoxicity, *etc.* (Haji et al., 1994; Park et al., 2000; Fang et al., 2007; Kang et al., 2010; Kim et al., 2014; Shin et al., 2016; Shin et al., 2017; Kang et al., 2018). Although there has been a lot of basic research on DHE, more clinical application experiments are needed based on the existing research to provide a sufficient scientific basis for their future use as effective drugs for AD and cerebrovascular diseases.

Previous work highlighted the role of DHE in the digestive system, which could ameliorate gastric injury in MNNG-induced CAG rats, IDO or glacial acetic acid-induced gastric ulcers (Wan and Bao, 2020; Wei et al., 2021; Wen et al., 2021). The results indicated that the therapeutic effects of DHE on CAG rats were presented in alleviating histological damage of gastric tissue *in vivo*, increasing cell proliferation of GES-1 cells, and ameliorating MNNG-induced gastric epithelial cell damage and mitochondrial dysfunction. In addition, DHE could inhibit MNNG-induced migration and invasion of GES-1 cells. It was found that DHE plays a crucial role in angiogenesis by inhibiting the HIF-1α-mediated VEGF pathway in CAG rats and gastric epithelial cells. Furthermore, DHE ameliorates dyspepsia, inflammatory infiltration, tissue damage, and serum oxidative stress through ERK/p38 and Rho/NF-κB signaling pathways. These studies provide a new promising therapeutic agent for the prevention and treatment of CAG and gastric ulcers. However, more animal studies and clinical trials are necessary to further confirm the protective and therapeutic effect of DHE on the digestive system.

In recent years, studies have found that DHE has cardiovascular and cerebrovascular system effects such as anti-arrhythmia, dilation of blood vessels, lowering blood pressure, slowing heart rate, inhibiting Ca^2+^ inflow, and selectively increasing cerebral blood flow (Schramm and Hamburger, 2014). DHE inhibits calcium overload-induced arrhythmias and works by prolonging the action potential duration in the cardiomyocytes of experimental animals (Baburin et al., 2018). It can reduce the amplitude and contractility of action potentials in human atrial and ventricular myocytes. Simultaneously, DHE (0.1, 0.3 μM) can reversibly and concentration-dependently reduce the influx of Na^+^ and Ca^2+^, and also inhibit atrial delayed depolarization caused by epinephrine and high extracellular Ca^2+^ in isolated human atrial and ventricular myocytes (Loh et al., 2014). Therefore, DHE can produce an anti-arrhythmic effect by reducing the inward current of Na^+^ and Ca^2+^, and increasing the pH value of intracellular fluid and Na^+^-H^+^ exchange in the resting state (Loh et al., 1992; Loh et al., 2014). In addition, DHE lowers blood pressure while slowing heart rate (Shoji et al., 1986; Yang et al., 1990). The related mechanisms of its anti-hypertensive effect include potassium channel activity (Chiou et al., 1996b).

**FIGURE 3:**
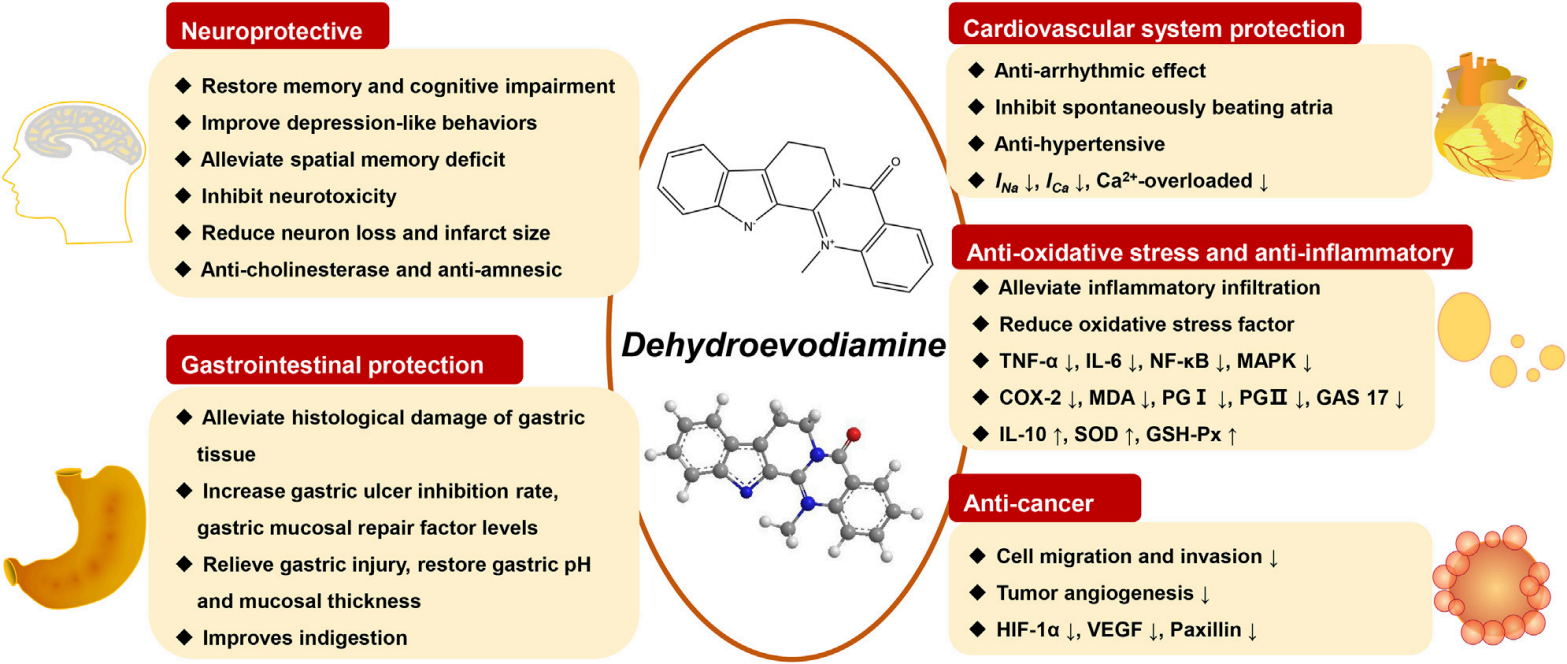
The pharmacological properties of DHE and its potential biological mechanisms.

DHE has a vasodilatory effect, which slows down the heart rate while reducing blood pressure. The lowering effect of diastolic blood pressure was stronger than that of systolic blood pressure, suggesting that DHE has the effect of dilating blood vessels (Loh et al., 1992). This vasodilator effect is related to calcium channel blockade, NO-cGMP system, potassium channel activity, *etc.* (Chiou et al., 1996b). In terms of two-way regulation of blood pressure, DHE has a clear blood pressure-lowering effect and a significant anti-hypertensive effect on diastolic blood pressure, which is manifested as vasodilation. Its vasodilatory mechanism is the inhibition of receptor-mediated Ca^2+^ channels and endothelial activation in vascular smooth muscle (Chiou et al., 1996b). The pharmacological properties of DHE and its potential biological mechanisms are shown in Figure 3.

Although the beneficial effects have been synthetically reported, the potential toxicological risks of DHE as a quinazoline alkaloid still need attention. In the cardiovascular system, studies have found that DHE has a proarrhythmic effect (Baburin et al., 2018; Hamburger, 2019), which can cause arrhythmias in chronic atrioventricular block dogs and in anesthetized rabbits. Specifically, DHE can prolong the QT interval of dogs and rabbits, and dogs can cause TdP. However, higher doses of DHE did not induce TdP. As for the digestive system, DHED was also reported to have potential hepatotoxicity (Lin et al., 2015). However, there was no significant difference in the liver/body weight ratio of mice after intragastric administration of large doses of DHE, suggesting that DHE did not cause significant hepatomegaly. In addition, 80 mg/kg DHE did not cause significant changes in serum liver function indicators ALT and AST levels in mice after 21 days of treatment, indicating that DHE had no significant hepatotoxicity. Moreover, it has also been reported that DHE destroys the homeostasis of bile acids without causing hepatotoxicity by upregulating CYP7A1 or BSEP through a mechanism that is yet to be determined (Zhang Y. et al., 2018). Thus, whether DHE causes hepatotoxicity varies based on experimental conditions and remains controversial. More rigorous and well-designed studies are needed to elucidate the toxicological risks of DHE.

Currently, most of the research on DHE is focused on its pharmacokinetics, chemical constituents, pharmacological effects, and mechanism of action. However, the research is superficial and not in-depth, and the research methods are relatively backward. Therefore, it is necessary to use advanced scientific and technological means to conduct more in-depth research on the chemical constituents and pharmacological mechanism of DHE, especially the pharmacological mechanism of the central nervous system, cardiovascular system, anti-tumor, anti-inflammatory, and other aspects. On the whole, the follow-up research on the chemical constituents and pharmacological activity of DHE should start from the following three aspects: ① The first is to deeply study its pharmacological effects and mechanism of action, and to more comprehensively expound the scientific connotation of the biological activity of DHE, which can provide a strong scientific basis for clinical application; ② The second is to explore the potential pharmacological effects of DHE that have not yet been reported, and expand the application scope of DHE; ③ Thirdly, strengthen the development and utilization of DHE, and enhance its effectiveness and give full play to its medicinal value by modifying the structure of DHE.

## Glossary

DHE: Dehydroevodiamine
TCM: Traditional Chinese medicine
EF: *Evodiae Fructus*
CVD: Cardiovascular diseases
LC-MS: Liquid chromatography-mass spectrometry
AD: Alzheimer’s disease
CAG: Chronic atrophic gastritis
RA: Rheumatoid arthritis
DMSO: Dimethyl sulfoxide
AChE: Acetylcholinesterase
AUC: Area under concentration-time curve
MRT: Mean residence time
CL: Clearance
BBB: Blood-brain barrier
ChEIs: Cholinesterase inhibitors
cx-DHED: Carboxy-dehydroevodiamine·HCl
NCAM: Neural cell adhesion molecule
CA: Calyculin A
PP: Protein phosphatase
Aβ: β-amyloid
SOD: Superoxide dismutase
p.o: Peros
i.p.: Intraperitoneal injection
ROS: Reactive oxygen species
GSK-3: Glycogen synthase kinase-3
WT/GFX: Wortmannin and GF-109203X
Tg: Transgenic
NMDARs: N-methyl-D-aspartate receptors
MCA: Middle cerebral artery
5-HT: 5-Hydroxytryptamine
NHE: Na^+^-H^+^ exchanger
IDO: Indomethacin
MPO: Myeloperoxidase
TNF-α: Tumor necrosis factor-α
IL-6: Interleukin-6
IL-10: Interleukin-10
ERK: Extracellular signal-regulated kinase
JNK: c-Jun N-terminal kinase
MNNG: N-methyl-N′-nitro-N-nitrosoguanidine
VEGF: Vascular endothelial growth factor
HIF-1α: Hypoxia-inducible factor-1 alpha
NF-κb: Nuclear factor kappa-B
TFF1: Trefoil factor family one
EGF: Epidermal growth factor
NO: Nitric oxide
IFN-α: Interferon alpha
LPS: Lipopolysaccharide
iNOS: Inducible nitric oxide synthase
CFA: Complete freund’s adjuvant
AIA: Adjuvant-induced arthritis
IL-1β: Interleukin-1 beta
IL-17: Interleukin-17
MMP-1: Matrix metallopeptidase 1
MMP-3: Matrix metallopeptidase 3
MAPK: Mitogen-activated protein kinase
CYP1A: Cytochrome P450 family 1 subfamily A member 1
hERG: Human ether-a-go-go related gene
TdP: Torsade de Pointes
EADs: Early after depolarizations
ALT: Alanine aminotransferase
AST: Aspartate aminotransferase
BSEP: Bile salt export pump
