# Supplementary material for: The pharmacokinetics profiles, pharmacological properties, and toxicological risks of dehydroevodiamine: A review
Source: Front Pharmacol. 2022 Nov 18;13:1040154. doi: 10.3389/fphar.2022.1040154 (PMC9715618; doi:10.3389/fphar.2022.1040154)
Supplement: Supplementary file 1 [file Table1.docx]

**Supplementary TABLE 1 The non-scientific name, class of name, and medicinal source of EF.**

| **Non-scientific name** | **Class of name** | **Medicinal source** |
| --- | --- | --- |
| *Euodia* | Other | Taiwan Herbal Pharmacop. 3rd Chinese ed. (MOHW, 2018) |
| *Euodia Fruit* | Other | Japanese Pharmacopoeia, 16th edn. (2012) |
| *Euodiae Fructus* | Pharmaceutical | Pharmacopoeia of China (2020)  Taiwan Herbal Pharmacop. 3rd Chinese ed. (MOHW, 2018)  Pharmacopoeia of China (2015)  Japanese Pharmacopoeia, 16th edn. (2012) |
| *Evodia* | Other | U.S. FDA Substance Registration System (2016)  Herbs of Commerce (McGuffin et al., 2000)  GRIN Report: World Economic Plants (Wiersema, 1999) |
| *Evodia Fruit* | Other | Korean Pharmacopoeia, 9th edn. (2007)  Japanese Pharmacopoeia 15th edn. (2006)  Illus. Chinese Materia Medica (Wu, 2005) |
| *Evodiae Fructus* | Pharmaceutical | Korean Pharmacopoeia, 9th edn. (2007)  Japanese Pharmacopoeia 15th edn. (2006) |
| *Fructus Evodiae* | Pharmaceutical | Hong Kong Chinese Materia Med. Standards (2014)  Illus. Chinese Materia Medica (Wu, 2005)  Pharmacopoeia of China (2005) |
| *Medicinal Euodia Fruit* | Other | Pharmacopoeia of China (2010) |
| *Medicinal Evodia* | Other | Medicinal Plants in China (WHO, 1997) |
| *Medicinal Evodia Fruit* | Other | Pharmacopoeia of China (2005) |
| *Osuyunamu* | Other | GRIN Report: World Economic Plants (Wiersema, 1999) |
| *Wu jhu yu* | Other | Taiwan Herbal Pharmacop. 3rd Chinese ed. (MOHW, 2018) |
| *Wu zhu yu* | Other | Taiwan Herbal Pharmacop. 3rd Chinese ed. (MOHW, 2018)  TCM Plants for Syst. Bio. Evaluation (Eisenberg et al., 2011)  Illus. Chinese Materia Medica (Wu, 2005)  Herbs of Commerce (McGuffin et al., 2000)  GRIN Report: World Economic Plants (Wiersema, 1999) |
| *Wuzhuyu* | Other | Med. Pls. Used by Maonan People in China. (Hong et al., 2015)  Pharmacopoeia of China (2015)  Hong Kong Chinese Materia Med. Standards (2014)  Trad. Med. in Dali Prefecture, SW China. (Zhang et al., 2014)  Pharmacopoeia of China (2010)  Pharmacopoeia of China (2005) |
| *Wúzhūyú* | Other | Medicinal Plants in China (WHO, 1997) |

*Notes: EF, Evodiae Fructus.*
